# Temporal analyses of germ cell mutations using the MutaMouse model support the recommended design in OECD test guideline 488

**DOI:** 10.1007/s00204-025-04085-1

**Published:** 2025-05-21

**Authors:** Gu Zhou, Andrew Williams, Danielle P. M. LeBlanc, George R. Douglas, Carole L. Yauk, Francesco Marchetti

**Affiliations:** 1 Environmental Health Science and Research Bureau, Healthy Environments and Consumer Safety Branch, 251 Sir Frederick Banting Driveway, Ottawa, Health CanadaON K1A 0K9 Canada; 2 Department of Biology, University of Ottawa, Ottawa, ON Canada

**Keywords:** Benzo[a]pyrene, *N*-ethyl-*N*-nitrosourea, Isopropyl methanesulfonate, Procarbazine, Benchmark dose

## Abstract

The Organisation for Economic Co-operation and Development test guideline (TG) 488 uses transgenic rodent models to assess in vivo mutagenesis. TG 488 recommends 28 consecutive days of exposure with sampling of germ cells from seminiferous tubules 28 days post-exposure (i.e., 28 + 28d). We analyzed mutant frequencies (MF) in male germ cells up to 70 days post-exposure to determine whether designs other than 28 + 28d are necessary for assessing germ cell mutagenicity. Adult MutaMouse males received various doses of benzo(a)pyrene (BaP), *N*-ethyl-*N*-nitrosourea (ENU), isopropyl methanesulfonate (iPMS), or procarbazine (PRC) alongside vehicle controls for 28 days orally. Germ cells were collected from seminiferous tubules at + 3d, + 28d, + 42d, or + 70d post-exposure and MF quantified using the *lacZ* assay. Significant increases in *lacZ* MF were observed for all four chemicals at 28 + 28d. No further increases occurred at later sampling times. There was no significant effect with BaP at 28 + 3d, and a significantly stronger response with ENU and BaP at 28 + 28d compared to 28 + 3d. PRC produced the strongest response at 28 + 3d, while there was no impact of different sampling times for iPMS. Both these chemicals significantly reduced testis weight at 28 + 3d and 28 + 28d. Finally, benchmark dose modeling generated overlapping confidence intervals among the four sampling times for ENU, iPMS, and PRC. However, for BaP, the confidence interval was significantly greater at 28 + 3d than at the other sampling times. These results support the use of the 28 + 28d design as the recommended experimental design for germ cells in TG 488 and that later sampling times are not necessary.

**Supplementary Information:**

The online version contains supplementary material available at 10.1007/s00204-025-04085-1.

## Introduction

Mutations, whether single nucleotide changes or chromosomal abnormalities, play a key role in birth defects and genetic diseases, including cancer (Ohno 2019; Rashed et al. 2022). Determining whether a chemical is capable of inducing mutations is an important aspect of regulatory evaluations. In vivo assays have a key role in assessing mutagenic hazard, because their outcome depends on in vivo metabolism, pharmacokinetics, and DNA repair processes. Among in vivo assays with an Organisation for Economic Co-operation and Development (OECD) test guideline (TG), the transgenic rodent (TGR) gene mutation assay 488 (TG 488) stands out as a critical tool (OECD 2022). The assay uses genetically modified rodents with chromosomally integrated plasmid or phage shuttle vectors containing bacterial reporter genes in every cell (Lambert et al. 2005). These reporter genes allow the detection and quantification of mutations across any tissue and cell type, except for female germ cells, since it is not possible to collect enough eggs to obtain sufficient DNA to conduct the assay (Yauk et al. 2005). The TGR assay can be seamlessly integrated with other genotoxic endpoints, enabling simultaneous analysis of micronuclei, Pig-a mutations, and DNA adducts (Lemieux et al. 2011; Long et al. 2016; Maurice et al. 2019). This assay has been used extensively for germ cell mutagenicity studies (Marchetti et al. 2018b). TGR models currently represent the most effective tools to evaluate the ability of chemicals to induce gene mutations in vivo for regulatory submissions.

DNA replication and cellular proliferation are crucial for converting chemically induced DNA lesions into stable mutations (Bielas and Heddle 2000). Gene mutation studies typically include a designated period after exposure to allow the manifestation of mutations (Thybaud et al. 2003). This period, often referred to as manifestation time, fixation time, expression time, or sampling time, is variable, because the rate of proliferation differs among tissues. Selecting an appropriate sampling time is particularly important for germ cells because of the length of spermatogenesis. In mice, for example, it takes about 49 days to generate mature sperm from spermatogonial stem cells (Adler 1996; Oakberg 1956). During this time, active proliferation is restricted to the spermatogonia compartment, and for the last 35 days of male germ cell development, there is no DNA replication. In contrast, the time required to generate mature hematopoietic cells is considerably shorter (Abramsson-Zetterberg et al. 1996); furthermore, hematopoietic cells replicate with an average doubling time of about 10 h (Pacchierotti et al. 1991, 2002). While it is accepted that each tissue may have an optimal sampling time based on its proliferation rate, it is neither practical nor desirable to have different sampling times for different tissues in regulatory testing primarily due to considerations related to cost and the 3R principles (Díaz et al. 2020).

OECD TG 488 describes the use of TGR models to generate in vivo mutagenicity data for regulatory decision-making, and specifies the recommended exposure duration and sampling time (OECD 2022). While a 28-day exposure, coupled with tissue sampling 3 days post-exposure (28 + 3d design), was initially recommended for rapidly proliferating tissues such as bone marrow, it was acknowledged that a longer sampling time may be preferable for slowly proliferating tissues such as liver (OECD 2011). The 28 + 3d design is also suboptimal for male germ cells, because a 3-day sampling time does not provide sufficient time for cells exposed during the proliferating phase of spermatogenesis, when mutations in the transgene can be induced, to populate the testis (Marchetti et al. 2018a). For example, short-term exposure studies with several established germ cell mutagens required sampling times longer than 3 days to detect a significant increase in germ cell mutations (Hachiya et al. 1999; Hoorn et al. 1993), with some requiring at least two weeks of sampling time before an effect could be observed (Hara et al. 1999; Suzuki et al. 1999). Moreover, using the recommended exposure regimen and sampling time from the 2013 guidance in TG 488 (OECD 2013), O’Brien and colleagues (O'Brien et al. 2016) showed that benzo(a)pyrene (BaP) does not increase mutant frequencies (MF) in germ cells from seminiferous tubules collected 3 days after the end of a 28-day exposure, while it does so in sperm collected 42 or 70 days later. Thus, identification of an optimal design for germ cell mutagenicity testing using TGR models requires consideration of the timing and processes of germ cell development.

Marchetti and colleagues (Marchetti et al. 2018a) simulated spermatogenesis to model the cellular composition of the mouse and rat testes, the duration of the various spermatogenic cell types, and their proliferating and DNA repair characteristics, to evaluate the impact of varying the sampling time on the ability to detect mutations using TGR models. These simulations supported the use of a 28 + 28d design as an acceptable design for both mice and rats. Specifically, the modeling made three predictions regarding the detection of mutations in germ cells using TGR models: (i) MF are expected to increase going from 28 + 3d to 28 + 28d; (ii) MF are expected to remain mostly stable at longer sampling times: and, (iii) the 28 + 28d design is an adequate experimental approach to determine whether a chemical is a germ cell mutagen in both mice and rats. Since TG 488 already recommends the 28 + 28d design for slow proliferating tissues such as liver (OECD 2013), and did not affect the qualitative or quantitative detections of mutations in a rapidly dividing tissue such as bone marrow (Douglas et al. 2022; Marchetti et al. 2021), data from the simulation of spermatogenesis suggest that the 28 + 28d study design could serve as a unifying regimen for assessing mutagenesis in any tissues. Indeed, the latest version of TG 488 (OECD 2022) recommends a common 28 + 28d design for both rapidly and slowly proliferating somatic tissues when both somatic and germ cell data are required.

A systematic empirical evaluation of the predictions from the simulation of rodent spermatogenesis is still lacking. Thus, we conducted a series of experiments to investigate the influence of sampling time on MF in male germ cells to provide empirical data in support of the prediction of the simulation of rodent spermatogenesis. Specifically, we exposed MutaMouse males to four established germ cell mutagens: BaP (O'Brien et al. 2016; Xu et al. 2014); *N*-ethyl-*N*-nitrosourea (ENU) (Katoh et al. 1994; O'Brien et al. 2015; Renault et al. 1997; Suzuki et al. 1997); isopropyl methanesulfonate (iPMS) (Katoh et al. 1994; Mattison et al. 1997); and procarbazine (PRC) (Suzuki et al. 1999). Each chemical was tested using a 28-day repeated-dose administration at various doses alongside concurrent controls. Male germ cells from seminiferous tubules were collected on sampling days + 3, + 28, + 42, or + 70 days. The *lacZ* assay was employed to determine MF, and Benchmark Dose (BMD) modeling was used to evaluate the effect of sampling time on mutagenic potency estimates.

## Materials and methods

### Chemicals

BaP (CAS 50–32-8; catalog no. B1760; powder, > 96% purity), ENU (CAS 759–73-9; catalog no. N8509; powder, ~ 53% purity), iPMS (CAS 926–06-7; catalog no. 808741; liquid, 99% purity), and PRC (CAS 366–70-1; catalog no. SML0036; powder, > 98% purity) were procured from Sigma-Aldrich Canada Co. (Oakville, Ontario, Canada). BaP and iPMS were dissolved weekly in olive oil obtained from Sigma-Aldrich. Both chemicals are stables at room temperature. PRC was prepared freshly using phosphate-buffered saline (PBS) without calcium and magnesium (Corning cellgro, Manassas, VA, USA), because it is sensitive to light. ENU was prepared freshly using pH 6.5 PBS and utilized within 2 h of preparation, because it is unstable and sensitive to light once dissolved.

### Animals and exposures

The use of mice in these studies was approved by the Health Canada Ottawa Animal Care Committee. All animal procedures and handling adhered to the guidelines set forth by the Canadian Council on Animal Care. The MutaMouse males used in these experiments were bred from an in-house colony, and housed under a 12-h light/12-h dark photoperiod at a room temperature of 21 °C and a relative humidity of 50%. Animals had ad libitum access to water and food throughout both the acclimation and experimental periods. The MutaMouse carries approximately 30 tandem copies of a recombinant λgt10 phage shuttle vector on each copy of chromosome 3 (Shwed et al. 2010); however, next-generation sequencing of the MutaMouse whole genome determined that at least one copy is not functional (Meier et al. 2019).

Adult MutaMouse males, aged between 9 and 14 weeks at the start of exposure, were randomly assigned to dose groups, typically with four mice in the control group and eight mice per treatment group per sampling time. Animals were housed individually in microVENT ventilated racks (Allentown, Allentown, NJ) with environmental enrichment. For each chemical, the highest dose was determined based on pilot dose range-finding studies aiming to exclude doses that induced excessive morbidity or toxicity, such as causing a 20% decrease in body weight (bw). The dose levels selected for the main studies were as follows: 0, 12.5, 25, 50, and 100 mg/kg bw/day for BaP; 0, 1, 2, and 5 mg/kg bw/day for ENU; 0, 1.25, 2.5, and 5 mg/kg bw/day for iPMS; and 0, 6.25, 12.5, and 25 mg/kg bw/day for PRC. The chemicals and vehicle controls were administered daily via oral gavage in a volume of 5 mL/kg bw for 28 consecutive days following TG 488. Animals were weighed daily and injected volume adjusted accordingly. The animals from the BaP, iPMS, and PRC experiments have already been used to evaluate the impact of sampling time on MF in bone marrow (Marchetti et al. 2021).

Animals were euthanized by cardiac puncture under isoflurane anesthesia at sampling times of + 3d, + 28d, + 42d, or + 70d. The testes were removed and weighed, and the extraction of male germ cells from seminiferous tubules followed a modified approach (O'Brien et al. 2014). Briefly, one end of the testis was held with forceps while a small hole was cut in the tunica albuginea at the opposite end using dissection scissors. The seminiferous tubules were then squeezed out through the incision, and the tunica was discarded. The procedure was repeated with the second testis. Next, 1 ml of Dulbecco’s PBS (D-PBS) was added to the decapsulated seminiferous tubules, and a tissue roller was gently moved back and forth across the tubules until they were flattened, and the D-PBS became cloudy with released cells. The cell suspension was collected into two 1.5 ml microfuge tubes, avoiding detached tubules, and the process was repeated but adding another 1 ml of D-PBS to the decapsulated seminiferous tubules. The final cell suspension had a total volume of 1 ml per tube. Cells were then centrifuged at 11,000 × g for 30 s, the supernatant was decanted, and pellets resuspended in 200 μl of D-PBS and combined. Then, cell suspension was aliquoted in individual tubes of 100 μl of D-PBS, frozen in liquid nitrogen, and stored at -80 °C until further analysis.

Three experiments were performed for BaP to generate the four sampling times. The first experiment involved doses from 0 to 50 mg/kg of BaP and did not include the + 28d sampling time. The second experiment used only 0 and 100 mg/kg of BaP dose and did not include the + 28d sampling time. The third experiment included all doses and just the + 28d sampling time. For ENU, one experiment included all doses and the + 3d and + 70d sampling times, while a second experiment included all doses and the + 28d and + 42d. Each experiment had its own concurrent controls. Conversely, all four sampling times for PRC and iPMS were part of single experiments where all animals were treated simultaneously and randomly allocated to one of the four sampling times.

### DNA extraction

Total genomic DNA from seminiferous tubule germ cells was extracted following the protocol outlined by O’Brien et al. (O'Brien et al. 2014). In brief, isolated germ cells were thawed and 100 μL of the sample were digested in 5 mL of lysis buffer containing 10 mM EDTA, 100 mM NaCl, 10 mM Tris–HCl (pH 7.6), 1% sodium dodecyl sulfate (v/v), and 1 mg/mL Proteinase K (Invitrogen, Burlington, Canada). This mixture was then incubated overnight at 37 °C with gentle shaking. Subsequently, genomic DNA was extracted using a phenol/chloroform extraction procedure and stored at 4 °C in 40–75 μL TE buffer (10 mM Tris pH 7.6, 0.1 mM EDTA) for further use.

### Mutant frequency analysis

The *lacZ* transgenes were rescued using a commercial packaging extract (Agilent Technology, Santa Clara, CA, USA) following the manufacturer's recommended conditions as described by Gingerich et al. (2014). MF were determined using the phenyl-β-D-galactopyranoside (P-gal)–positive selection assay (Lambert et al. 2005). In brief, phage particles were adsorbed to Escherichia coli C *lacZ– galE–* host cells and selected for growth on LB/saline minimal agar plates containing 0.3% phenyl β-D-galactoside (P-gal; Sigma-Aldrich, Oakville, ON, Canada). Concurrently, bacteria were plated on nonselective minimal agar to enumerate total plaque-forming units (pfu) or titer. All plates were then incubated overnight at 37 °C. The MF was expressed as the ratio of mutant plaques to total pfu. Since no tissue nor DNA was available, MF for the BaP exposed groups at 28 + 3d are those previously reported (O'Brien et al. 2016).

### Statistical analyses

We used the ANOVA test followed by Tukey's Honest Significant Difference (HSD) test to assess the significance of changes in testis weight between control and treated groups. The test adjusts p values for multiple comparisons considering the number of dose groups, the degrees of freedoms, and the significant threshold for pairwise comparisons.

Typically, two-to-four *lacZ* reactions were required to reach the minimum number of pfu for each animal as per TG 488. Thus, we first used a generalized linear mixed model (GLMM) with a binomial error distribution in R software (version 4.3.2; https://www.r-project.org/), considering dose effects and random individual differences, to identify potential outlier replicates. With this approach outliers are defined as observations with model residuals exceeding a cut-off point, typically > 3.5 standard deviations of the mean (Gelman and Hill 2007); no outliers were found in our data. Then, the data were aggregated by animal, and analyses were repeated to identify individual animal outliers. This led to the elimination of five animals out of 456 (1.1%). Among these, one animal was a control at 28 + 28d in the BaP experiment, two were in the iPMS experiment (one at 1.25 mg/kg/day at 28 + 3d and another at 28 + 28d at 2.5 mg/kg/day), and two were in the PRC experiment (one at 6.25 mg/kg/day at 28 + 42d and the other at 25 mg/kg/day at + 28d). These five animals in indicated in red in Supplementary Data.

Estimated MF were obtained using generalized linear models with the quasibinomial distribution in R software. The Wald statistic was used to analyze MF changes among treatment groups across all time points. Pairwise comparisons were performed using the doBy R library and estimates were back-transformed (Højsgaard and Halekoh 2018). The delta method approximated the back-transformed standard errors of the estimated MF. Finally, a Holm–Sidak correction was applied to adjust p values for multiple comparisons. This method sequentially adjusts p values based on their rank while accounting for the number of comparisons to control the family-wise error rate.

### Benchmark dose modeling

PROASTweb 70.1 software (https://proastweb.rivm.nl/) was used for benchmark dose (BMD) modeling. A likelihood ratio test was used to test the null hypothesis that the response did not differ among dose levels, followed by a Bartlett test to assess equal variance of log-transformed response data. Four mathematical models (Hill, exponential, reverse exponential, and lognormal) were applied to fit the dose–response data. The exponential model with the lowest Akaike Information Criterion (AIC) was selected as the best fitting model. The BMD combined-covariate approach was employed to examine the influence of sampling time on the potency (i.e., BMD) of each tested chemical (Wills et al. 2016). The benchmark response (BMR) was set to a 50% increase in response over the modeled control group (BMD50) as recommended (White et al. 2020), and time points were set as covariates. The lower and upper 90% confidence limits of BMD50 (BMDL and BMDU) were used to evaluate the effect of sampling time.

## Results

### Assessment of chemical toxicity

**Fig. 1:**
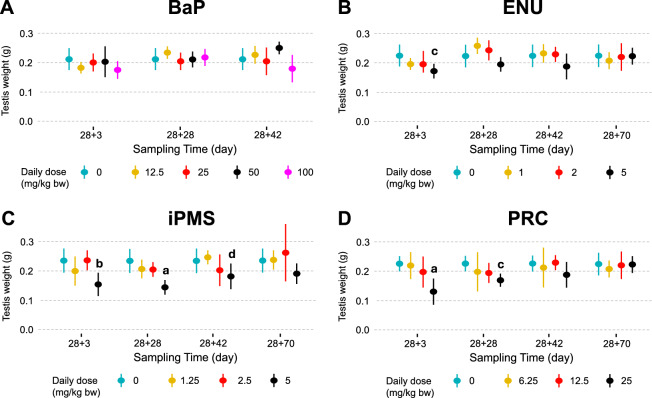
Combined testis weight at necropsies in MutaMouse animals exposed to benzo(a)pyrene (**a**), *N*-ethyl-*N*-nitrosourea (**b**), isopropyl methanesulfonate (**c**), and procarbazine (**d**) at the four sampling times. For each dose group, the mean ± standard deviation is shown. Testis weights were not recorded at 28 + 70d for BaP because of scale failure. Statistical comparisons versus concurrent controls of each chemical and sampling time are presented. The level of significance is indicated as follows: a = p < 0.0001; b = p < 0.001; c = p < 0.01; d = p < 0.05. Controls = turquoise; low dose = yellow; medium dose = red; high dose = black; BaP extra high dose = purple

We used testis weight changes after chemical exposure to assess germ cell toxicity. Testis weights were generally lower at the early sampling times followed by full or partial recovery at the 70d sampling time (Fig. 1; Supplementary Data). No changes in testis weights were observed for BaP for the first three sampling times (Fig. 1A). Testis weights could not be recorded at 28 + 70d because of a malfunctioning scale. For ENU, testis weights decreased by 23.8%, 13.0%, 16.2%, and 1.0% at 5 mg/kg/day at 28 + 3d, 28 + 28d, 28 + 42d, and 28 + 70d, respectively. Significant decreases (p < 0.01) only occurred at 28 + 3d compared to controls (Fig. 1B). Testis weight decreased by 34.7%, 38.6%, 22.7%, and 19.5% at 28 + 3d, 28 + 28d, 28 + 42d, and 28 + 70d, respectively, after exposure to iPMS at 5 mg/kg/day with statistically significant decrease at 28 + 3d (p < 0.001), 28 + 28d (p < 0.0001) and 28 + 42d (P < 0.05), with respect to controls (Fig. 1C). iPMS is the only chemical for which testis weight had not yet fully recovered at 28 + 70d. Finally, PRC induced the largest decrease in testis weight with losses of 41.1%, 24.9%, 20.6%, and 8.2% at 28 + 3d, 28 + 28d, 28 + 42d, and 28 + 70d, respectively, with a statistically significant decrease observed at 28 + 3d (p < 0.0001) and 28 + 28d (p < 0.01) compared to controls (Fig. 1D).

### Analyses of mutant frequencies in tubule germ cells from control animals by age

**Fig. 2:**
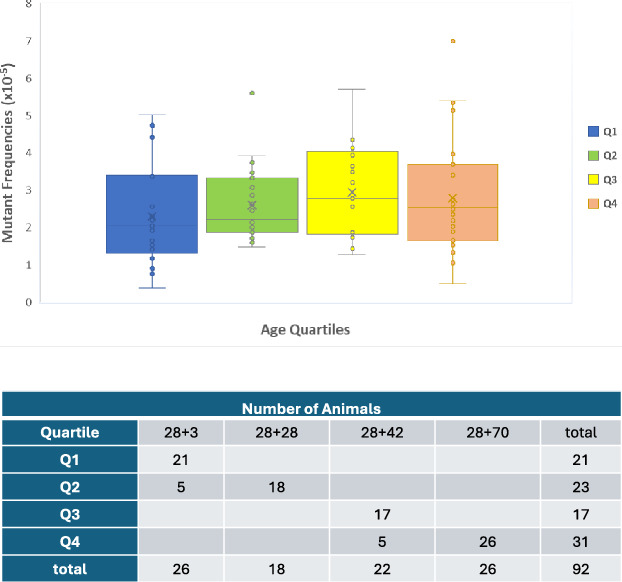
Mutant frequencies in control animals by age. The top panel shows the mutant frequencies in control animals by age quartile. The lines in the middle of quartile boxes represent the median MF of the quartile; the x in the middle of quartile boxes is the average of MF of the quartile. The bottom panel shows the distribution of control animals from the four sampling times among quartiles

We analyzed a total of 92 unexposed MutaMouse males across the four chemical exposures (Table 1). Among these, 26, 18, 22, and 26 mice were allocated to controls at 28 + 3d, 28 + 28d, 28 + 42d, and 28 + 70d, respectively. The age range spanned from 13 to 28 weeks at necropsy and no significant changes in MF were detected across ages (p = 0.404). The background level of MF (× 10^–5^ ± standard deviation) at 28 + 3d, 28 + 28d, 28 + 42d, and 28 + 70d were 2.3 ± 1.2, 2.7 ± 1.1, 3.1 ± 1.5, and 2.6 ± 1.2, respectively. However, since the animals' ages varied at the start of each experiment, we also analyzed spontaneous MF by separating the animals in quartiles based on the age at euthanasia. As depicted in Fig. 2, the median MF (× 10^–5^) in each quartile was 2.0, 2.2, 2.8, and 2.5, respectively. Statistical analysis revealed no significant differences across quartiles (p = 0.227) (Fig. 2). These results do not show an age-related increase in spontaneous MF over the covered age range. Thus, we merged controls from all four sampling times within each chemical experiment for the statistical analyses of mutagenic effects.

**Table 1:** *LacZ* mutant frequencies in tubule germ cells of MutaMouse control mice aggregated by sampling time

Sampling times (days)	No. of animals^a^	No. of mutants	No. of Pfu^b^	Average of MF (× 10^–5^)	SD
28 + 3	26 (11, 7, 4, 4)	217	8,992,221	2.3	1.2
28 + 28	18 (8, 4, 3, 3)	192	6,912,110	2.7	1.1
28 + 42	22 (11, 4, 4, 3)	281	9,005,142	3.1	1.5
28 + 70	26 (11, 7, 4, 4)	236	9,637,491	2.6	1.2

a: Numbers in parenthesis are the control animals from the experiments with benzo(a)pyrene, *N*-ethyl-*N*-nitrosourea, isopropyl methanesulfonate, and procarbazine, respectively

b: Plaque-forming units

### Qualitative analyses of *lacZ* mutant frequencies across sampling times in male germ cells

#### Benzo[a]pyrene

**Fig. 3:**
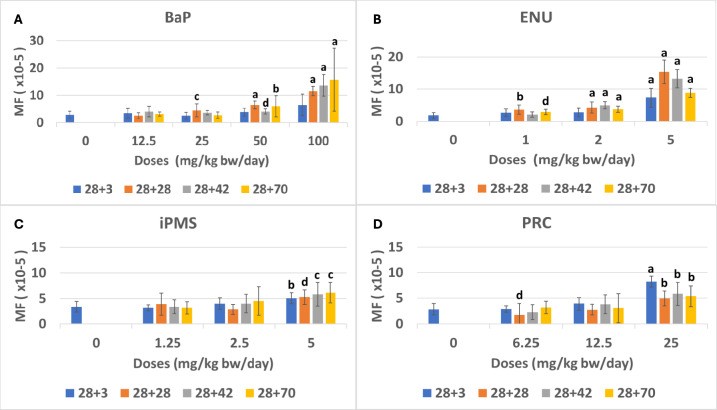
*LacZ* mutant frequencies (MF) in tubule germ cells of MutaMouse males exposed to benzo(a)pyrene (**a**), *N*-ethyl-*N*-nitrosourea (**b**), isopropyl methanesulfonate (**c**), and procarbazine (**d**) at 28 + 3d (blue), 28 + 28d (orange), 28 + 42d (gray), and 28 + 70d (yellow). Mean ± standard deviation is shown for each dose group. Controls at all sampling times were combined into a single control group (blue bar) for dose–response analysis across time point for each chemical tested. Note that the Y axis is not the same for each panel. Statistical significance with respect to concurrent controls is indicated for each dose and sampling time. Significance levels are denoted as follows: a = p < 0.0001; b = p < 0.001; c = p < 0.01; and d = p < 0.05

Dose-dependent increases in *lacZ* MF (× 10^–5^ ± SD) were observed at all sampling times but 28 + 3d following BaP exposure (Table 2, Fig. 3A). At this sampling time, MF reached a maximum of 6.4 ± 3.9 at the high-dose group of 100 mg/kg bw/day BaP. However, this 2.3-fold increase above controls did not achieve statistical significance (p = 0.07) because of the large intra-group variability. The two highest BaP doses produced significant increases at all other sampling times reaching a maximum MF of: 11.6 ± 1.7 (4.2-fold above control; p < 0.0001) at 28 + 28d; 13.6 ± 3.9 (4.9-fold above controls; p < 0.0001) at 28 + 42d; and, 15.7 ± 11.5 (5.7-fold above controls; p < 0.0001) at 28 + 70d for 100 mg/kg/day. A significant increase in MF with 25 mg/kg/day was observed only at 28 + 28d (4.5 ± 2.4; 1.6-fold above controls; p < 0.01), while no significant effect was observed with 12.5 mg/kg/day BaP, regardless of sampling time. These results are in accordance with the MF predictions of the modeling of spermatogenesis and support the selection of the 28 + 28d design as the optimal design for germ cell mutagenicity testing with the *lacZ* assay.

**Table 2:** LacZ mutant frequencies in tubule germ cells of MutaMouse mice exposed to benzo(a)pyrene and collected at various sampling times

Sampling time (days)	Dose (mg/kg bw/day)	No. of animals^a^	No. of mutants	No. of Pfu^b^	MF^c^ (× 10^–5^)	SD^d^ (× 10^–5^)	Fold change	adj-p^e^
28 + 3	0	41	410	15,309,394	2.77	1.37		
	12.5	5	52	1,452,396	3.44	1.69	1.24	0.72
	25	5	38	1,449,083	2.56	1.22	0.92	1.00
	50	6	85	2,173,044	3.87	1.46	1.40	0.29
	100	6	102	2,341,199	6.45	3.95	2.33	0.07
28 + 28	0	41	410	15,309,394	2.77	1.37		
	12.5	8	63	2,490,625	2.52	1.11	0.91	1.00
	25	8	109	2,412,928	4.47	2.39	1.61	** < 0.01**
	50	8	152	2,361,403	6.44	1.44	2.32	** < 0.0001**
	100	6	219	1,894,062	11.58	1.71	4.18	** < 0.0001**
28 + 42	0	41	410	15,309,394	2.77	1.37		
	12.5	6	108	2,659,276	4.03	1.95	1.45	0.05
	25	6	105	2,793,796	3.69	0.82	1.33	0.16
	50	6	140	3,411,238	4.06	0.94	1.46	** < 0.05**
	100	5	244	1,823,325	13.61	3.95	4.91	** < 0.0001**
28 + 70	0	41	410	15,309,394	2.77	1.37		
	12.5	6	76	2,439,604	3.09	0.76	1.11	0.96
	25	6	80	2,635,751	2.74	1.15	0.99	0.98
	50	5	159	2,524,095	6.00	3.85	2.16	** < 0.001**
	100	4	234	1,416,945	15.71	11.54	5.67	** < 0.0001**

a: Combined controls from all four sampling times. See Table 1

b: Plaque-forming units (Pfu)

c: Average mutant frequencies (MF)

d: Standard deviation (SD) of the arithmetic mean of individual animals

e: Adjusted p values vs controls (Bold p values indicate statistical significance after Holm–Sidak correction for multiple comparisons)

#### N-ethyl-N-nitrosourea

Exposure to ENU caused dose-dependent increases in *lacZ* MF at all four sampling times in tubule germ cells of MutaMouse males (Table 3, Fig. 3B). At 28 + 3d, only the high dose of ENU induced a statistically significant increase in MF (7.3 ± 2.9; 4.0-fold above controls; p < 0.0001). At 28 + 28d and 28 + 70d, all doses induced statistically significant increases in MF, reaching a maximum of 15.3 ± 3.7 (8.4-fold above controls; p < 0.0001) and 8.8 ± 1.4 (4.8-fold above controls; p < 0.0001) at the high dose. At the low dose, ENU induced increases in MF to 3.7 ± 1.4 (2.0-fold above controls; p < 0.001) and 2.9 ± 0.8 (1.6-fold above controls; p < 0.05) at 28 + 28 and 28 + 70, respectively. Finally, at 28 + 42d, only the top two doses induced significant increases in MF of 13.2 ± 2.9 (7.2-fold above controls; p < 0.0001) and 5.0 ± 1.1 (2.7-fold above controls; p < 0.001). The ENU results also support the model of spermatogenesis and the selection of the 28 + 28d design as the optimal design for germ cell mutagenicity testing with the *lacZ* assay.

**Table 3:** LacZ mutant frequencies in tubule germ cells of MutaMouse mice exposed to *N*-ethyl-*N*-nitrosourea and collected at various sampling times

Sampling time (days)	Doses (mg/kg bw/day)	No. of animals^a^	No. of mutants	No. of Pfu^b^	MF^c^ (× 10^–5^)	SD^d^ (× 10^5^)	Fold changes	adj-p^e^
28 + 3	0	22	134	7,000,078	1.83	0.90		
	1	6	92	3,006,351	2.74	1.23	1.49	0.50
	2	6	47	1,629,993	2.82	1.34	1.53	0.25
	5	6	210	2,640,888	7.33	2.91	3.99	** < 0.0001**
28 + 28	0	22	134	7,000,078	1.83	0.90		
	1	8	92	2,367,873	3.70	1.43	2.02	** < 0.001**
	2	8	123	2,666,400	4.31	1.68	2.35	** < 0.001**
	5	7	352	2,272,113	15.34	3.66	8.36	** < 0.0001**
28 + 42	0	22	134	7,000,078	1.83	0.90		
	1	8	47	4,776,206	2.15	0.87	1.17	0.85
	2	8	148	4,912,046	5.03	1.06	2.74	** < 0.001**
	5	8	356	5,099,254	13.19	2.89	7.19	** < 0.0001**
28 + 70	0	22	134	7,000,078	1.83	0.90		
	1	7	70	2,270,625	2.95	0.80	1.61	** < 0.05**
	2	7	112	2,869,017	3.83	0.95	2.09	** < 0.001**
	5	7	195	2,175,034	8.82	1.43	4.81	** < 0.0001**

a: Combined controls from all four sampling times. See Table 1

b: Plaque-forming units (Pfu)

c: Average mutant frequencies (MF)

d: Standard deviation (SD) of the arithmetic mean of individual animals

e: Adjusted p values vs controls (Bold p values indicate statistical significance after Holm–Sidak correction for multiple comparisons)

#### Isopropyl methanesulfonate

iPMS induced a consistent statistically significant increase in MF across sampling times only for the high dose of 5 mg/kg bw/day (Table 4, Fig. 3C). MF were stable across sampling times. In fact, fold changes for the high-dose group ranged between 1.5 and 1.8 depending on the sampling time, with the MF reaching a maximum of 6.1 ± 2.0 (p < 0.01) at 28 + 70d. No other statistical increases in MF were observed with respect to controls at any of the other doses investigated regardless of sampling time. The results with iPMS show no significant impact of sampling time on MF and, thus, do not fully align with the predictions of the model of spermatogenesis.

**Table 4:** LacZ mutant frequencies in tubule germ cells of MutaMouse mice exposed to isopropyl methanesulfonate and collected at various sampling times

Sampling time (days)	Doses (mg/kg bw/day)	No. of animals^a^	No. of mutants	No. of Pfu^b^	MF^c^ (× 10^–5^)	SD^d^ (× 10^–5^)	Fold change	adj-p^e^
28 + 3	0	15	204	6,229,380	3.39	1.08		
	1.25	7	84	2,656,290	3.14	0.61	0.93	0.99
	2.5	8	130	3,205,474	3.98	1.16	1.17	0.18
	5	7	144	2,812,683	5.05	1.06	1.49	** < 0.001**
28 + 28	0	15	204	6,229,380	3.39	1.08		
	1.25	8	121	3,074,267	3.89	2.18	1.15	0.52
	2.5	7	71	2,501,723	2.84	1.02	0.84	0.84
	5	8	156	3,061,176	5.27	1.44	1.55	** < 0.05**
28 + 42	0	15	204	6,229,380	3.39	1.08		
	1.25	8	94	2,821,790	3.37	1.47	1.00	1.00
	2.5	8	123	3,159,584	3.98	1.82	1.17	0.67
	5	7	147	2,569,976	5.82	2.29	1.72	** < 0.01**
28 + 70	0	15	204	6,229,380	3.39	1.08		
	1.25	8	104	3,338,834	3.13	1.23	0.92	099
	2.5	8	148	3,114,192	4.49	2.83	1.33	0.15
	5	8	217	3,515,438	6.14	2.03	1.81	** < 0.01**

a: Combined controls from all four sampling times. See Table 1

b: Plaque-forming units (Pfu)

c: Average mutant frequencies (MF)

d: Standard deviation (SD) of the arithmetic mean of individual animals

e: Adjusted p values vs controls (Bold p values indicate statistical significance after Holm–Sidak correction for multiple comparisons)

#### Procarbazine hydrochloride

Similar to iPMS, PRC induced a consistent statistically significant increase in MF across sampling times only for the high dose of 25 mg/kg bw/day (Table 5, Fig. 3D). Fold changes for the high-dose group ranged between 1.7 and 2.9 depending on the sampling time. Surprisingly, the highest MF (8.2 ± 1.5; p < 0.0001 versus controls) was observed at 28 + 3d and the lowest response (4.9 ± 0.4; p < 0.001 versus controls) was observed at 28 + 28d. The differences at the highest dose between 28 + 3d and other sampling times were statistically significant (p < 0.001, p < 0.05, p < 0.01, respectively). There were no other statistically significant increases in MF observed with respect to controls at any of the other doses investigated regardless of sampling time. Thus, as for iPMS, the results with PRC show no qualitative impact of sampling time on MF and do not align with the predictions for MF based on the model of spermatogenesis.

**Table 5:** LacZ mutant frequencies in tubule germ cells of MutaMouse mice exposed to procarbazine and collected at various sampling times

Sampling time (days)	Doses (mg/kg bw/day)	No. of animals^a^	No. of mutants	No. of Pfu^b^	MF^c^ (× 10^–5^)	SD^d^ (× 10^–5^)	Fold change	adj-p^e^
28 + 3	0	14	178	6,008,052	2.83	1.18		
	6.25	8	87	2,949,855	2.88	0.560	1.02	1.00
	12.5	8	114	2,954,988	3.88	1.27	1.37	0.19
	25	8	198	2,452,851	8.21	1.55	2.90	** < 0.0001**
28 + 28	0	14	178	6,008,052	2.83	1.18		
	6.25	6	64	3,472,032	1.76	0.43	0.62	0.15
	12.5	6	110	4,048,056	2.75	0.32	0.97	0.82
	25	6	140	2,817,155	4.91	0.44	1.74	** < 0.001**
28 + 42	0	14	178	6,008,052	2.83	1.18		
	6.25	7	56	2,456,000	2.26	0.96	0.80	0.50
	12.5	8	118	3,061,511	3.78	1.31	1.39	0.58
	25	6	155	2,658,947	5.82	1.17	2.06	** < 0.001**
28 + 70	0	14	178	6,008,052	2.83	1.18		
	6.25	7	78	2,440,925	3.19	0.86	1.13	0.96
	12.5	7	81	2,678,491	3.05	0.87	1.08	0.99
	25	8	145	2,808,705	5.35	1.73	1.89	** < 0.001**

a: Combined controls from all four sampling times. See Table 1

b: Plaque-forming units (Pfu)

c: Average mutant frequencies (MF)

d: Standard deviation (SD) of the arithmetic mean of individual animals

e: Adjusted p values vs controls (Bold p values indicate statistical significance after Holm–Sidak correction for multiple comparisons)

#### Impact of sampling time

Table 6 provides a qualitative summary of the doses at which there were significant increases in MF relative to controls over the four sampling times for the four chemicals studied. Consistent with our previous model of induced mutagenesis during spermatogenesis, the results show that false-negative results can be obtained using the 28 + 3d design (i.e., no statistically significant effect with BaP). We also find that study designs with sampling times longer than 28 + 28d do not confer additional sensitivity to detect an effect and may even result in reduced sensitivity (i.e., the high dose of BaP and the low dose of ENU induced statistically significant increases only at 28 + 28d). Overall, these qualitative results support the use of the 28 + 28d design as the preferred design for assessing germ cell mutagenicity using the TGR assay.

**Table 6:** Summary of doses with statistically significant increases in *lacZ* mutant frequencies with respect to controls by sampling time

Chemical	Sampling time			
	28 + 3	28 + 28	28 + 42	28 + 70
BaP	–	M, H, EH	H, EH	H, EH
ENU	H	L, M, H	M, H	L, M, H
iPMS	H	H	H	H
PRC	H	H	H	H

L = low, M = medium, H = high, EH = extra high

### Quantitative analyses: BMD modeling to examine the effect of sampling time on mutagenic potency

**Fig. 4:**
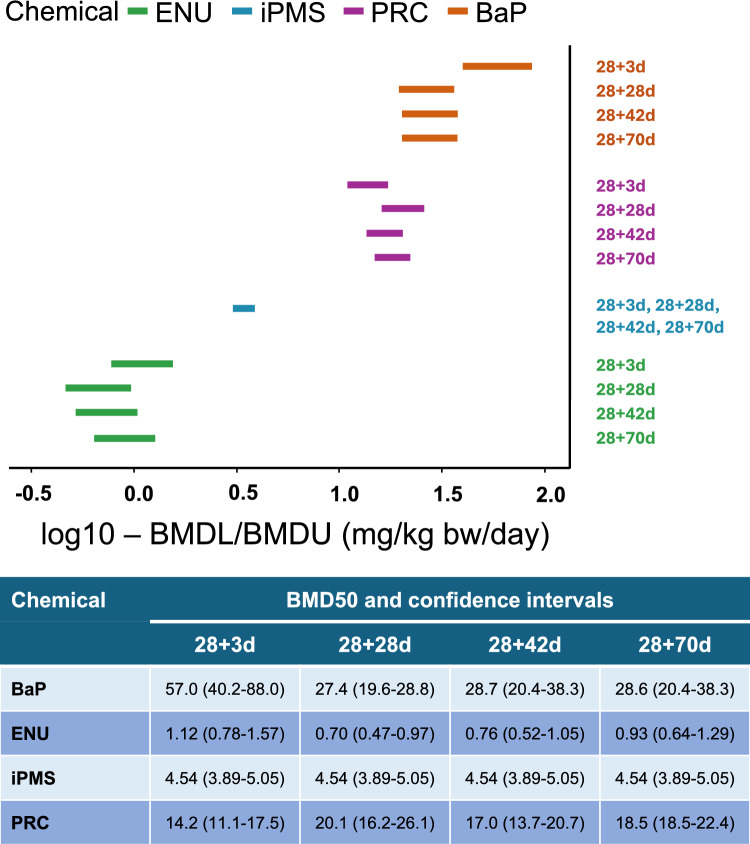
Log10 benchmark dose (BMD) for a 50% increase in *lacZ* mutations at the four sampling times. 90% confidence intervals based on exponential model with the lowest Akaike Information Criteria (AIC) using PROAST are shown. Confidence intervals are organized from lowest (top) to highest (bottom) potency. BMD50 and BMDL/U values are shown in the bottom panel

To explore the impact of sampling time on chemical potency estimates, we modeled the dose–response data for each chemical by fitting exponential functions to calculate BMDs and their corresponding 90% confidence intervals (BMDL/BMDU). For each chemical, the four mathematical models generated very consistent BMD values at each sampling time (Supplementary Fig. 1). Figure 4 reports the BMD_50_ and confidence intervals from the exponential model with the best AIC value. Distinct BMD_50_ values (mg/kg/day) were determined for each sampling time for BaP, ENU, and PRC. For BaP, the BMD confidence interval at 28 + 3d (40.2 – 88.0 mg/kg/day) was significantly higher than the confidence intervals obtained for the other three sampling times (Fig. 4). This higher confidence interval suggests that BaP’s potency in producing a 50% increase in MF above background is underestimated using the 28 + 3d design. Conversely, overlapping BMD confidence intervals were observed for ENU and PRC at the four sampling times (Fig. 4). Finally, a single dose–response curve adequately fits the data for all four sampling times for iPMS (Fig. 4), resulting in a BMD_50_ of 4.54 mg/kg/day (90% CI: 3.89—5.05 mg/kg/day). This did not change when using a different mathematical model (Supplementary Fig. 1). Overall, these analyses show that, except for BaP, the sampling time did not have a major impact on chemical potency values.

## Discussion

We investigated the influence of sampling time on MF in male germ cells of MutaMouse animals exposed to four established mutagens to provide empirical data on the use of the 28 + 28d design as the optimal experimental protocol for germ cell mutagenicity testing with TGR models. We found that: (i) positive results can be obtained with the 28 + 3d design; (ii) a negative result at 28 + 3 does not exclude the possibility that the tested chemical is a germ cell mutagen (i.e., potential for false negatives); (iii) for strong mutagens, the 28 + 28d design provides greater sensitivity than the 28 + 3d design than other sampling times; and (iv) sampling times longer than 28 + 28d are not necessary. Overall, the results support the use of the 28 + 28d design as the recommended experimental protocol in OECD TG 488.

The results with BaP and ENU are aligned with the prediction of the spermatogenesis model that MF are expected to increase following mutagen exposures at 28 + 28d with respect to 28 + 3d and should not appreciably change thereafter. Of note, extending the sampling time to + 28d results in a qualitative change for BaP, as no statistically significant effect was observed at the earlier sampling time. Therefore, BaP would be incorrectly classified as negative for germ cell mutagenesis if tested only at 28 + 3d. These findings reflect the fact that most germ cells collected from seminiferous tubules at 28 + 3d (i.e., spermatids) have received the exposure during periods of germ cell development with no DNA synthesis and/or cell division (Marchetti et al. 2018a). This makes it unlikely to produce mutations that can be detected with the TGR assay (Bielas and Heddle 2000). Extending the sampling time from + 3d to + 28d allows time for these non-proliferating germ cells to move out of the testis. It also allows for exposed stem cells to populate the testis with developing germ cells that have sustained DNA damage during periods of active DNA and cell replication increasing the chances of fixing transgene mutations.

Additional support for the fact that the 28 + 28d study design confers greater sensitivity to detect mutagenic effects comes from the observation that the high dose of BaP and low dose of ENU induced significant increases in MF only at this time point. As discussed previously (Marchetti et al. 2018a), the analysis of tubule germ cells within the 28 + 28d design allows the assessment of effects in a population that has sustained part of the exposure as differentiating spermatogonia rather than just as stem cell spermatogonia. Since the former population divides more rapidly than the latter (Adler 1996; de Rooij 2001), they undergo more cell divisions during the exposure period resulting in a greater chance for mutations to be induced and fixed in the transgene. This is also reflected in the lowest BMD (i.e., 27.4 and 0.70 mg/kg/day) being observed at 28 + 28d for both BaP and ENU, respectively. Thus, the 28 + 28d design provides higher sensitivity to detect chemically induced mutations than the other sampling times.

The results with iPMS and PRC are not aligned with the prediction of the spermatogenesis model (Marchetti et al. 2018a). In fact, the results show no impact of sampling time on MF for iPMS, while for PRC, there is a significant decline in MF going from 28 + 3d to 28 + 28d; however, MF at 28 + 28d are still significantly higher than controls for both chemicals. Of particular note, these two chemicals were the most toxic to spermatogenesis: PRC-exposed mice had the highest reduction in testis weight in this study (i.e., 40% for the high dose at 28 + 3d); while iPMS is the only chemical for which testis weight had not yet fully recovered at 28 + 70d, suggesting that it had significantly and permanently reduced the pool of stem cell spermatogonia. Studies with several DNA damaging agents have shown that cell killing is occurring mostly in differentiating spermatogonia, stem cells, and, to a lesser extent, spermatocytes (Meistrich et al. 1982). Following injury, stem cells favor regeneration over differentiation with surviving stem cells repopulating tubule sections that have lost stem cells (Klein et al. 2010; Yoshida 2012). We speculate that the unexpected results obtained with the *lacZ* assay are the consequence of the strong cytotoxic effects exerted by these two chemicals on spermatogenesis, which were not considered in the spermatogenesis modeling (Marchetti et al. 2018a). Specifically, we posit that as spermatids present in the tubules at the beginning of the exposure moved out of the testis, they were not fully replaced by newly formed cells that would have been derived from the killed spermatogonia. This resulted in the dramatic decline in testis weight observed at 28 + 3d and in a testis enriched for germ cells where mutations could be induced. Nevertheless, the observed results with PRC and iPMS, do not negate that the 28 + 28d design by itself is sufficient to properly characterize the germ cell mutagenicity of chemicals, even those that are highly cytotoxic to spermatogenesis.

The application of BMD modeling is gaining acceptance in determining chemical-specific potency (White et al. 2020; Wills et al. 2016). Our BMD combined-covariate approach provides further support for the use of the 28 + 28d design as the preferred experimental design for germ cell mutagenicity testing. BMD confidence intervals were overlapping across timepoints for all chemicals except BaP (only one BMD interval was obtained for iPMS), indicating that potency estimates were not impacted by sampling time. However, for BaP, the confidence intervals were higher for the 28 + 3d design, indicating that this design is prone to underestimating potency relative to 28 + 28d. Thus, our results support that the 28 + 28d design is also preferable for chemical potency analysis using BMD modeling.

All four chemicals induced significant increases in mutations at 28 + 70d. The time lapse between the end of exposure and sampling time exceeds the established duration of spermatogenesis in mice (Marchetti et al. 2018a). Therefore, mutations measured at this sampling time represent mutations that have occurred in stem cell spermatogonia. Although the magnitude of increase in germ cells is lower that the magnitude of effect observed in bone marrow (Marchetti et al. 2021) for the three chemicals in common, that is BaP, iPMS, and PRC, these results show that chemical exposure can impact spermatogonial stem cells and induce a permanent increase in germ cells carrying mutations that can be potentially transmitted to the next generation.

An increase in germ cell mutations as function of age is well established in humans (Goldmann et al. 2019; Kong et al. 2012; Sasani et al. 2019; Shojaeisaadi et al. 2024). However, contrasting results have been observed using rodent TGR models. Hill et al. reported no increase in mutations in the germ cells of 2-year-old BigBlue mice (Hill et al. 2004), while a significant increase in mutations was observed in *lacI* mice of similar age (Walter et al. 1998). Here, analysis of *lacZ* mutations in a set of 92 controls animals that spanned an age difference of ~ 100 days between the youngest and oldest mice did not show a significant increase in MF. Our results suggest that the age difference in this study is not sufficient to observe an effect with the *lacZ* assay and analyses in older animals would be required to detect a significant increase in age-related germ cell mutations in mice.

In summary, the results of this study support the use of the 28 + 28d as the preferred design for assessing germ cell mutagenicity using TGR mouse models. Thus, the 28 + 28d design provides a unifying approach for assessing mutagenicity in any tissue, thereby contributing to reducing the number of animals required for regulatory testing. Data in rats supporting these findings would build further confidence on the use of this experimental design to generate robust mutagenicity data for regulatory submissions independently of the TGR model used.

## Supplementary Information

Below is the link to the electronic supplementary material.

Supplementary file1 (XLSX 52 KB)

## Data Availability

Testis weights and mutant frequency data for individual animals are provided in Supplementary Data.

## Code availability

PROAST in R (version 70.3, https://rivm.nl/en/proast).
